# Midkine and CA9 Are Potential Cancer Stem Cell Biomarkers in Triple‐Negative Breast Tumours

**DOI:** 10.1155/ancp/3265988

**Published:** 2026-08-30

**Authors:** Jorge Choque, Aparicio Aguilar, Javier Enciso-Benavides, Nancy Rojas-Moran, Nathaly Enciso, Carlos Castañeda Altamirano, Miluska Castillo, Luis Alfaro, Javier Enciso

**Affiliations:** ^1^ Regenerative Medicine Group, Scientific University of the South, Lima, Peru; ^2^ Faculty of Biology, National University of San Marcos, Lima, Peru, unmsm.edu.pe; ^3^ Stem Cell Laboratory, Enciso Veterinary Clinic, Lima, Peru; ^4^ Electron Microscopy Laboratory, Faculty of Medicine, National University of San Marcos, Lima, Peru, unmsm.edu.pe; ^5^ National Institute of Neoplastic Diseases, Lima, Peru

**Keywords:** biomarker, CA9, cancer stem cells, midkine, secretome, triple-negative breast cancer

## Abstract

**Introduction:**

There is no specific therapy for triple‐negative breast cancer (TNBC), and the recurrence rate is high. Cancer stem cells (CSCs) play an important role in cancer chemoresistance and metastasis, but there is no consensus marker in this type of cancer. The aim of this study was to identify CSC biomarkers in the plasma secretome of TNBC patients.

**Methods:**

CD44+/CD24− CSCs were isolated from MDA‐MB‐436 and MDA‐MB‐231 (ATCC) lines by magnetic immunoselection. The extracellular vesicles (EVs) of CSCs were isolated by size exclusion chromatography (SEC) of conditioned medium (CM) without fetal bovine serum (FBS) and blood plasma from TNBC and healthy women. Tetraspanins CD9 and CD81 identified the EVs, while electron microscopy determined the morphology and tunable resistive pulse sensing (TRPS) established particle size. Luminex technology was used to determine the presence of L1 cell adhesion molecule (L1CAM), carbonic anhydrase IX (CA9), mesothelin, midkine, hepsin, kallikrein‐6 (KLK6), transglutaminase 2 (TGM2), aldehyde dehydrogenase 1a1 (ALDH1A1), epithelial cell adhesion molecule (EpCAM), and differentiation cluster 44 (CD44).

**Results:**

Particles of 0–200 nm in size were more frequent in CSC‐MDA‐MB‐436, while particles of 201–500 nm were more frequent in CSC‐MDA‐MB‐231. In blood plasma, the particle size was 150–250 nm in TNBC patients and 150–300 nm in healthy individuals. Particle concentrations were 1.3 × 10^10^ in CSCs of the MDA‐MB‐231 (CSC‐MDA‐MB‐231) cell line and 3.8 × 10^7^ in the MDA‐MB‐231 cell line (L‐MDA‐MB‐231). The concentration of CSCs in MDA‐MB‐436 was 2.7 × 10^8^ compared to 9.4 × 10^7^ in L‐MDA‐MB‐436. Midkine and CA9 were present in the EVs of both CSC lines and in the plasma of women with TNBC but absent in the blood plasma of healthy women. Midkine showed better statistical discrimination.

**Conclusions:**

The size and concentration of EVs are heterogeneous. Midkine and CA9 are produced by CSCs in women with TNBC and may be possible biomarkers of these cells in this tumour type.

## 1. Introduction

Nonluminal triple‐negative breast cancer (TNBC) does not express progesterone, oestrogen, or HER2 receptors [1]. This type of cancer responds very well to initial chemotherapy, but recurrence is high [2], and there is a high level of heterogeneity and apparent plasticity that contributes to its aggressiveness, making treatment difficult [3]. It has been reported that cell subpopulations in TNBC are differentiated and have the capacity of self‐renewal, with both characteristics having been postulated as essential for drug resistance, metastasis, and tumour recurrence [4, 5].

These subpopulations called cancer stem cells (CSCs) with a CD44+/CD24− immunophenotype are present in higher numbers in this type of cancer compared to luminal and HER2 phenotypes [6]. They are also highly enriched in mouse models of breast cancer lung metastasis [7] and are tumourigenic [8], suggesting that these cells may play a role in tumour invasion and metastasis [9]. Thus, these cells should be targeted to develop a potential treatment plan to overcome cancer and prevent resistance and relapse [10].

In vitro studies of CSCs with the CD44+/CD24− immunophenotype from two TNBC cell lines have shown that one of these lines has a higher proliferative capacity and ultrastructurally differed from tumour cells with another immunophenotype, inferring that their secretomes would also be different [11, 12]. Secretomes contain extracellular vesicles (EVs) with a nonreplicating bilayer membrane structure that all cells can secrete and release outside [13]. They carry abundant inclusions, such as nucleic acids, proteins, and lipids, which play a key role in intercellular communication [4]. Tumour‐derived EVs are involved in different stages of metastasis, including angiogenesis [14]. A recent study proposed that exosomal vesicles can transmit information from CD63‐positive neoplastic cells in Luminal‐A and oestrogen receptor–positive breast tumours, while high levels of differentiation cluster 44 (CD44) are associated with short survival and the triple‐negative phenotype, and their plasma levels correlate with the cancer burden of breast cancer patients [15], both of which are related to CSCs.

On the other hand, the secretome of CSCs greatly influences the development of TNBC, and thus, many new antitumour therapies are focused on targeting these communication networks to eradicate the tumour and prevent metastasis, tumour relapse, and drug resistance [16].

The development of new personalised antitumour therapies for cancer patients highlights the importance of the CSC secretome in tumour development, chemoresistance, and relapse, as new therapies must combine different epithelial‐mesenchymal transition and CSC inhibitors. Robust biomarkers are needed to identify patients who will benefit from these treatments [16, 17].

The aim of this study was to explore the presence of a common cancer–related molecule that could be a specific marker for CSCs in the secretome of plasma from women with TNBC and two lines of CSC TNBC. This would allow the determination of the relevance of these cells in the different stages of the evolution of this cancer and evaluate whether they could be a target for chemotherapy in metastasis and tumour recurrence.

## 2. Materials and Methods

### 2.1. Cells

This study involved work with the MDA‐MB‐436 (ATCC HTB‐130) and MDA‐MB‐231 (ATCC HTB 26) cell lines, both of which are human TNBC lines of the claudin‐low transcriptional subtype (ATCC, USA) [18]. These cell lines were maintained and expanded in DMEM‐F‐12 culture medium (Sigma) supplemented with 4.5% glucose, 4 mM L‐glutamine, 1% antibiotics (Merck Millipore), 1% essential amino acids (MEM, Sigma), and 10% fetal bovine serum (FBS). In addition, the MDA‐MB‐436 cells were also supplemented with 0.01 mg/mL insulin (Sigma) and 16 mcg/mL glutathione (Sigma) [12]. The EVs produced by the cell lines were used as a positive control for the proteins present in the serum of women with TNBC. This is because these cell lines are already characterised and known to produce the proteins studied, so they could be isolated and then compared with those found in the patients’ plasma.

### 2.2. Immunoselection of Cells

The protocol used in this study established two stages of immunomagnetic cell selection using the MiniMACS magnetic separator (Miltenyi Biotec), as described elsewhere [12]. In brief, after reaching 70% cell confluence, cells were detached with 0.025% trypsin, centrifuged at 300 g, and concentrated to a total of 10^7^ cells. They were then labelled with CD24‐biotin and CD44‐MACS MicroBeads (Miltenyi) antibodies to mark the target cells.

The first step was negative selection to obtain a CD24− cell population using an LD column (Miltenyi). Then, cells from the CD24− population were subjected to positive selection by passage through LS columns (Miltenyi) for the magnetic capture of cells with a CD44+ phenotype. A population of cells with the CD44+/CD24− phenotype was thus obtained and corroborated by flow cytometry, as described below [12]. In brief, this was performed with a Guava Easy‐Cyte (Merck/Millipore) using CD24‐PE (MACS Cod. 130‐098‐861) and CD44‐FITC (Abcam ab95138) monoclonal antibodies on cell cultures from the 2nd–5th passage after thawing. Cells with this phenotype were seeded in 175 cm^2^ flasks (SPL Life Sciences Co., Ltd., Korea) and expanded in an incubator (Nuve Incubator, EC 160, Turkey) at 37°C, 5% CO_2_, and 90% humidity before being cryofrozen at −170°C in liquid nitrogen.

### 2.3. Conditioned Medium (CM)

CM was obtained from the proposed and immunoselected lines (CSC) in this work, considering three isolates for each cell type. After reaching a minimum of 80% confluence in Flask 175 (Falcon), the culture medium was changed to a similar medium but without FBS. After 48 h, the medium was removed and centrifuged at 1500 rpm for 5 min to remove cell debris from the pellet. The supernatant was frozen at −80°C.

### 2.4. Blood Plasma

Blood was collected from the cephalic vein in EDTA tubes (BD Vacutainer Blood) from 19 women diagnosed with TNBC before neoadjuvant/adjuvant therapy, with anatomical staging as follows: two patients with stage IA, one with stage IIA, five with stage IIIA, five with stage IIB, four with stage IIIB, and 2 with stage IV and 12 healthy women who were in physical health and, in particular, had no tumours. Following collection, the plasma was frozen at −80°C. Informed consent was obtained previously from both the women with TNBC and the healthy participants (Letter Number 065‐2019‐CRPI‐DI‐DICON/INEN). The study was approved by the Ethics Committee of the National Institute of Neoplastic Diseases (Resolution Number 282‐2019.CIEI/INEN).

### 2.5. Isolation and Characterisation of the Secretome

#### 2.5.1. Isolation

Size exclusion chromatography (SEC) with qEV10/35 nm columns (Izon) was used to purify the EVs. The supernatant of the CM and blood plasma (15 mL) was thawed and then concentrated using Amicon Ultra‐15 centrifugal filters (Sigma–Aldrich, MO, USA) with a molecular weight cut‐off of 30 kDa and centrifuged at 5000 g for 30 min following the manufacturer’s instructions. The concentrated material (250 µL) was manually separated into sterile microtubes and then separated into different solutions for quantitative particle analysis.

On the other hand, taking into account previous work showing different protein concentrations in blood serum, in the present study, total proteins were quantified from the secretome isolated from the blood plasma of women with TNBC and healthy women. Each isolated fraction was quantified using the Broad Range Protein Assay Kit (Invitrogen) using the Qubit 2.0 (Invitrogen), following the manufacturer’s instructions.

#### 2.5.2. Characterisation

Characterisation of CSC EVs from the two cell lines and blood plasma was performed by detecting particle size, morphology, and identity using EV surface markers. In the case of blood plasma, the amount of total proteins in women with TNBC and in healthy women was also determined.

##### 2.5.2.1. Secretome Particle Size Analysis

We studied particles from chromatographic fraction 8 of each blood plasma sample and fractions 6, 7, and 8 of the previously concentrated conditioned media, which were obtained from triple‐negative breast tumor cell lines (MDA‐MB‐231 and MDA‐MB‐436), and from immunoselected cells with the CD44+/CD24− immunophenotype (CSC‐ MDA‐MB‐231 and CSC‐MDA‐MB‐436), and from blood plasma of TNBC and healthy women.

High‐precision measurements of the physicochemical properties of EVs, such as size and concentration, were performed by tunable resistive pulse sensing (TRPS) using the qNano equipment (Izon Science), according to the protocol established by the manufacturer of the qNano equipment and the Control Suite Software (Izon Science).

qNano works based on the Coulter principle, whereby two fluid cells containing the sample to be analysed (upper cell) and the electrolyte (lower cell) are separated by an insulating membrane (the nanopore). As the particles have a higher resistance than the electrolyte, when passing through the pore, they momentarily reduce the current passing through the pore, generating a blockage, which is the resistive pulse that shows the volume of the particle by grouping the data of the magnitude and duration of each blockage. On the other hand, the number of times these blockages are generated determines the concentration of the particles. To achieve this, pressure is applied, which in this study was 9 mbar. The frequency of blockages is linearly scaled with the pressure applied, providing a dispersion concentration, which is shown as the particle concentration using the Izon qNano Control Suite software.

Given the wide possibilities of proteins within the secretome analysed, the existence of multiple pleiotropic bioactive compounds is understood. Therefore, computer filtering was conducted using the Izon Control Suite software, whereby the concentration of particles is quantitatively separated into three different groups, classified into small, medium, and large particles (0–200; 201–500; and >500, respectively) to be able to establish differences and contrast them with each other.

##### 2.5.2.2. Morphology of Vesicles

To determine the morphology of the vesicles, the protocol described by Soares et al. [19] was used. In brief, freshly prepared secretomes were isolated by transmission electron microscopy (TEM). Fractions 6, 7, and 8 of the chromatographic elutions for the cell‐CM samples and fraction 8 of the blood plasma samples were concentrated. They were then eluted in 100 μL of phosphate‐buffered saline (PBS) and then fixed with 100 μL of 4% paraformaldehyde (final concentration of 2% paraformaldehyde). In all cases, 20 μL of EV preparations was allowed to adsorb in a 75 mesh Formvar/carbon‐coated grid for 30 min at room temperature. The grids were then washed with PBS (membrane side down) and dried with filter paper. For negative staining, the EV grids were transferred with a drop of 50 μL of 3% phosphotungstic acid solution (pH 7) for 10 min and then dried with filter paper. TEM visualisations were performed with a FEI TECNAI G2 SPIRIT BIOTWIN transmission electron microscope from the National Institute of Children’s Health (INSN), and images were captured with a slow scan charged couple device (CCD) camera.

##### 2.5.2.3. Identity

Western blotting of secretomes from triple‐negative breast tumour cell lines and blood plasma from women with TNBC and healthy women was performed to demonstrate the presence of CD9, one of the most specific and widely expressed markers for extracellular membrane vesicles [20], which together with tetraspanin CD81 are highly representative of EVs in the breast cancer cell line [21].

The protocol described by Soares et al. [19] was used with minor modifications and is briefly explained below. Loading buffer with β‐mercaptoethanol and 2% SDS was added to the isolated secretomes. The preparations were then boiled at 90°C for 30 min, separated on a 4%–10% SDS‐polyacrylamide gel gradient (SDS‐PAGE), and electrophoretically transferred to nitrocellulose membranes. The membranes were blocked in 5% nonfat dry milk powder in 1× TBS‐T (0.5% Tween‐20) at room temperature and incubated overnight at 4°C with the following primary antibodies: anti‐CD9 (1:2000) (ab236630; abm) and anti‐CD81 (1:1000) (SC166029; Santa Cruz Biotechnology). These are commonly used EV markers. The membranes were then washed with 1× TBS‐T (0.5% Tween‐20). The secondary antibodies used for CD9 and CD81 were goat antirabbit pAb (1:5000) (IgG horseradish peroxidase [HRP]) (ab7090; abm) and goat antimouse pAb (1:1000) (IgG HRP) (ab9740; abm), respectively. Secondary antibodies were incubated for 2 h, with shaking at room temperature. HRP chemiluminescence was detected using Western blot enhanced chemiluminescence substrate (Thermo Fisher Scientific), and signals were captured by X‐ray.

### 2.6. Cancer Marker Identification

To identify possible cancer markers, the commercial Milliplex MAP Human Circulating Cancer Biomarker Magnetic Bead, Panel 4 (Millipore, HCCB4MAG‐58K) kit was used, which contains recognised markers for several types of cancer, but which have not been studied in TNBC stem cell EVs. The molecules studied were L1 cell adhesion molecule (L1CAM), carbonic anhydrase IX (CA9), mesothelin, midkine, hepsin, kallikrein‐6 (KLK6), transglutaminase 2 (TGM2), aldehyde dehydrogenase 1a1 (ALDH1A1), epithelial cell adhesion molecule (EpCAM), and differentiation cluster 44 (CD44), with their respective controls.

The principle of the technique is that the sample, which in this case was the secretome of the cells studied, and blood plasma from TNBC and healthy women, is added to a mixture of colour‐coded beads precoated with analyte‐specific capture antibodies. The antibodies bind to the analytes of interest. Biotinylated detection antibodies specific to the analytes of interest are added and form an antibody‐antigen sandwich complex.

All the antibodies bound to streptavidin were read on the Luminex (Millipore) MAGPIX luminescence instrument, a fluorescence‐based instrument using CCD imaging technology. The xMAP beads are a family of fluorescently stained carboxylated polystyrene microspheres that act both on the surface for the solution‐phase assay and with the spectral identifier detected by the instrument.

### 2.7. Statistical Analysis

The general results were observed by means of histograms, and the system indicates the standard deviation (SD) for each reading. The results were analysed using the software programme GraphPad Prism version 6. Kolmogorov–Smirnov and Shapiro–Wilk tests were used to assess the normal distribution of the data obtained. The particle diameter sizes of the cell line and CSCs were assessed by the Mann–Whitney and Kruskal–Wallis tests. Comparison between the concentration and particle diameter of the cell line and CSCs was assessed by ANOVA. A *p*‐value < 0.05 was considered significant.

## 3. Results

### 3.1. Isolation and Characterisation of the Secretome

Following the protocol described in Section 2, populations of cells with CD44+/CD24− immunophenotype considered CSCs were isolated by magnetic immunoselection, corroborated by flow cytometry, which verified that the MDA‐MB‐436 line had a purity of 87.6% and the MDA‐MB‐231 line 80.4%. Then, FBS‐free CM was then obtained from the cultured cell lines and CSCs. Subsequently, the secretome was isolated from the cell cultures and blood plasma following the protocol described in Section 2 and cryopreserved at −80°C.

#### 3.1.1. Total Protein in the Blood Plasma Secretome

**Figure 1:**
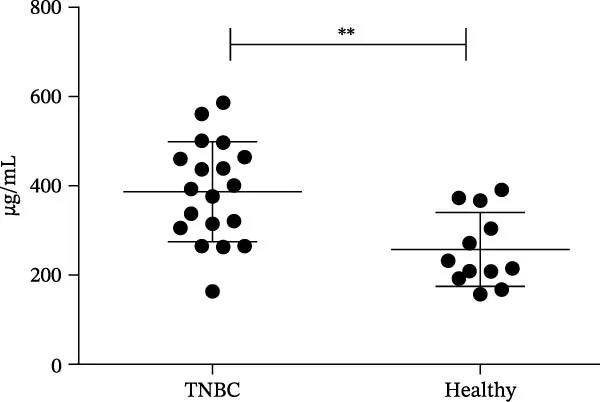
Total protein concentrations of TNBC patients (*n* = 19) and patients without a history of cancer (*n* = 12).  ^∗∗^ *p* < 0.01.

A statistically significant difference (*p*  < 0.01) was observed between the amount of total protein in the secretome of patients with TNBC (median = 393 µg/mL) and that of healthy individuals without a history of cancer (median = 223.7 µg/mL) (Figure 1).

#### 3.1.2. Secretome Particle Size Analysis

##### 3.1.2.1. Cell Line Secretome Particle Size

CM was obtained from the CSCs and tumour cells of each cell line studied, prior to the isolation of their corresponding EVs. Then, using qNano equipment (Izon) and TRPS technology, five readings were performed, applying 9‐mbar pressure for both secretomes derived from tumor cell lines (MDA‐MB‐231 and MDA‐MB‐436) and cells with CD44+/CD24− (CSC) immunophenotype from MDA‐MB‐231(CSC‐MDA‐MB‐231) and MDA‐MB‐436 (CSC‐MDA‐MB‐436) lines. The data obtained in these multiple readings of their secretome did not vary, which allowed proceeding to quantitative analysis of the reading with the most intermediate results between all readings of each secretome for both the MDA‐MB‐231 and MDA‐MB‐436 cell lines, their tumor cells, and the CD44+/CD24− (CSC) immunophenotype. All sample readings reached a minimum particle count of 500 particles, as recommended by the qNano supplier to obtain statistically representative data to determine concentration and size.

Given the wide possibility of proteins within the secretome and consequently multiple pleiotropic bioactive compounds, computer filtering with the Izon Control Suite software was used, allowing the particles to be quantitatively separated into three different groups of small, medium, and large particles (0–200; 201–500; and >500, respectively) to establish differences for comparison with the current literature on nomenclature according to their size.

**Figure 2:**
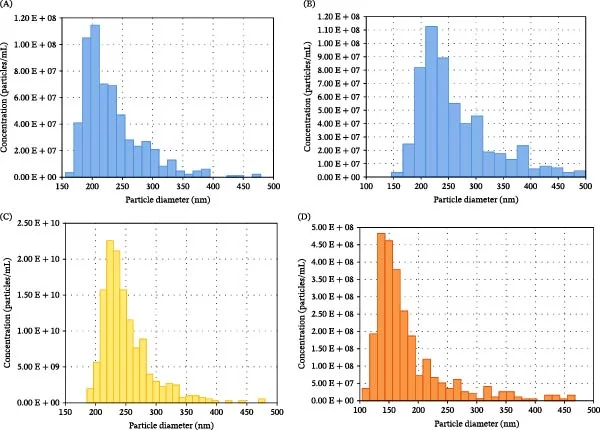
Secretome particle size. (A) Cell line MDA‐MB‐231. (B) Cell line MDA‐MB‐436. (C) CSC‐MDA‐MB‐231 (CD44+/CD24− phenotype). (D) CSC‐MDA‐MB‐436 (CD44+/CD24− phenotype).

The average particle size obtained from the MDA‐MB‐231 secretome was a concentration (particle/mL) of 3.26 × 10^7^, with a mean diameter of 226 nm and a SD of 46.6 (Figure 2A). The average particle size obtained from the MDA‐MB‐436 secretome was a concentration (particle/mL) of 1.29 × 10^8^, with a mean diameter of 258 nm and a SD of 53.9 (Figure 2B). On the other hand, the CSC‐MDA‐MB‐231 secretome had an average concentration (particle/mL) of 1.30 × 10^10^, with a mean diameter of 250 nm and a SD of 41.3 (Figure 2C). The average obtained from the CSC‐MDA‐MB‐436 secretome was a concentration (particle/mL) of 2.64 × 10^8^, with a mean diameter of 182 nm and a SD of 60.6 (Figure 2D).

##### 3.1.2.2. Differences in Particle Size Between Cell Lines and Their CSCs

Quantitative differentiated analyses were performed according to the concentration of particles with respect to the size range for each cell line and CSC. This was obtained using the Izon Control Suite software, separating all particles obtained from the secretome of the cell lines mentioned above into three different groups. These groups were characterised as follows: from 0 to 200 nm (small), 201–500 nm (medium), and larger than 501 nm (large).

**Figure 3:**
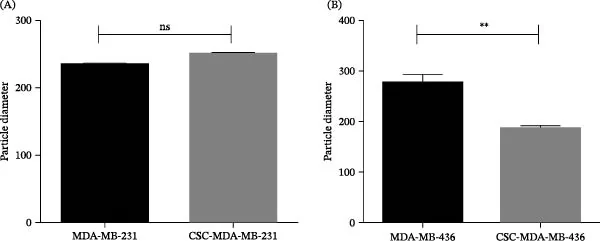
Comparison between EV diameter of MDA‐MB‐231 and CSC‐MDA‐MB‐231 (A) and MDA‐MB‐436 and CSC‐MDA‐MB‐436 (B). Data represent the mean ± SD.  ^∗∗^ represents *p* < 0.01 and ns, not significant. *n* = 3 different samples.

No significant difference (*p* = 0.1) in size was observed between the nanoparticles of the MDA‐MB‐231 and the secretome of CSC‐MDA‐MB‐231 (Figure 3A). The mean diameter of EVs derived of MDA‐MB‐231 was 234 nm (±SD 52.9), while that of the CSC‐MDA‐MB‐231 was 250 nm (SD 41.3). On the other hand, the mean diameter of EVs of MDA‐MDA‐MB‐436 was 291 nm (+SD 52.9), while that of CSC‐MDA‐MB‐436 was 190 nm (+SD 87.4), showing a significant difference (Figure 3B).

Significant differences were observed in the analysis of particle diameter and concentration with respect to the size range for each sample of each cell line.

**Figure 4:**
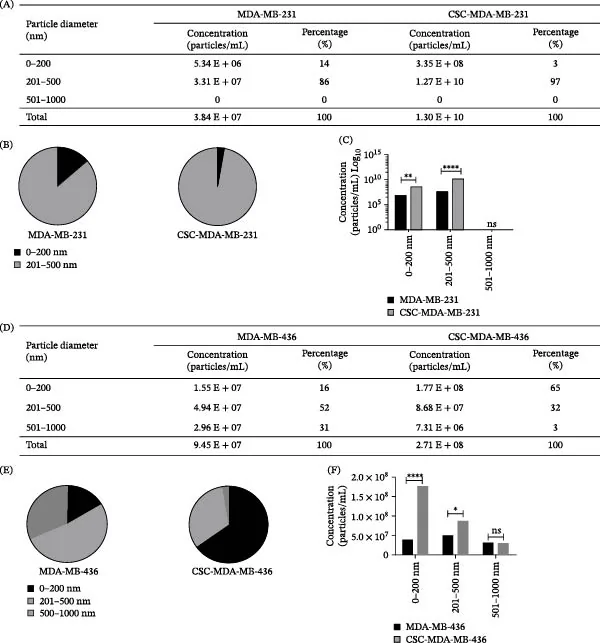
(A–C) Differences between MDA‐MB‐231 and CSC‐MDA‐MB‐231 secretomes. (A) Frequency of particles differentiated by concentration according to size range. (B) Graph of frequency by size range. (C) Statistical analysis between the secretome of MDA‐MB‐231 and CSC‐MDA‐MB‐231 (*n* = 3). Data represent the mean ± SD.  ^∗∗^ *p* < 0.01;  ^∗∗∗∗^ *p* < 0.0001; and ns, not significant. (D–F) Differences between MDA‐MB‐436 and CSC‐MDA‐MB‐436 secretomes. (D) Frequency of particles differentiated by concentration according to size range. (E) Graph of frequency by size range. (F) Statistical analysis between the secretome of MDA‐MB‐436 and CSC‐MDA‐MB‐436 (*n* = 3). Data represent the mean ± SD.  ^∗^ *p* < 0.05;  ^∗∗∗∗^ *p* < 0.0001; and ns, not significant.

With respect to the MDA‐MB‐231 cell line, the highest frequency of particles for both analyses of the secretome samples ranged from 201 to 500 nm (MDA‐MB‐231: 86% and CSC‐MDA‐MB‐231: 97%). Meanwhile, a higher concentration of nanoparticles was found in the secretome of CSC‐MDA‐MB‐231 than that of MDA‐MB‐231 (MDA‐MB‐231: 38 million/mL and CSC‐MDA‐MB‐231: 13 billion/mL) (Figure 4A–C).

On the other hand, the frequency of particles in the secretome of MDA‐MB‐436 was higher, ranging from 201 to 500 nm (42%), whereas the particles in the secretome of CSC‐MDA‐MB‐436 showed the highest frequency, ranging from 0 to 200 nm (65%). However, the concentration of particles was higher in the secretome of CSC‐MDA‐MB‐436 than MDA‐MB‐436 (MDA‐MB‐436: 117 million/mL and CSC: 271 million/mL) (Figure 4D–F).

##### 3.1.2.3. Plasma Secretome Particle Size

**Figure 5:**
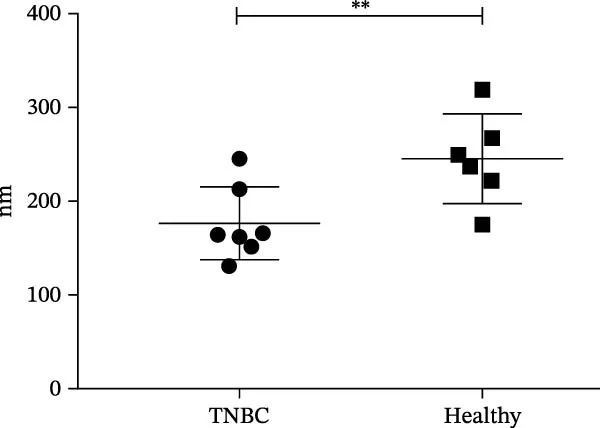
Particle diameter in patients with TNBC and healthy individuals.  ^∗∗^ *p* < 0.01.

SEC is the recommended technique for the isolation of EVs from plasma, as it achieves the highest efficiency and least damage to the vesicles [22, 23], and very little protein remains in the fraction. Particle analysis using the qNano, system based on TRPS, revealed a particle size of 150–250 nm in samples from patients with TNBC and 150–300 nm in healthy individuals (Figure 5).

#### 3.1.3. Morphology

**Figure 6:**
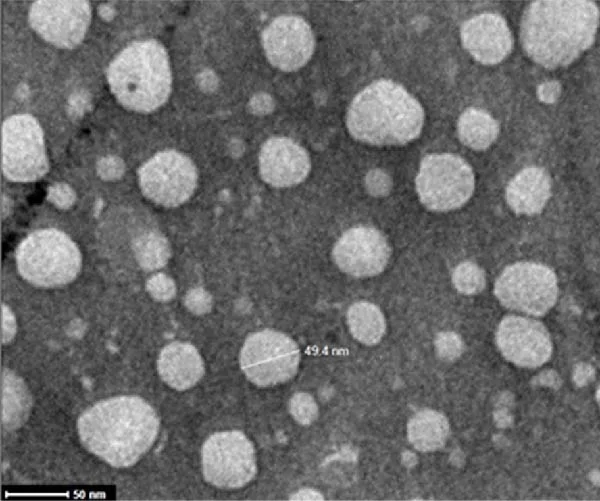
Image showing the rounded shape and size heterogeneity of CD44+/CD24− cancer stem cell EVs. Transmission electron microscopy.

With the use of TEM, EVs derived from CD44+/CD24− CSC presented a spherical image and a size of 30–500 nm (Figure 6).

#### 3.1.4. Identity

**Figure 7:**
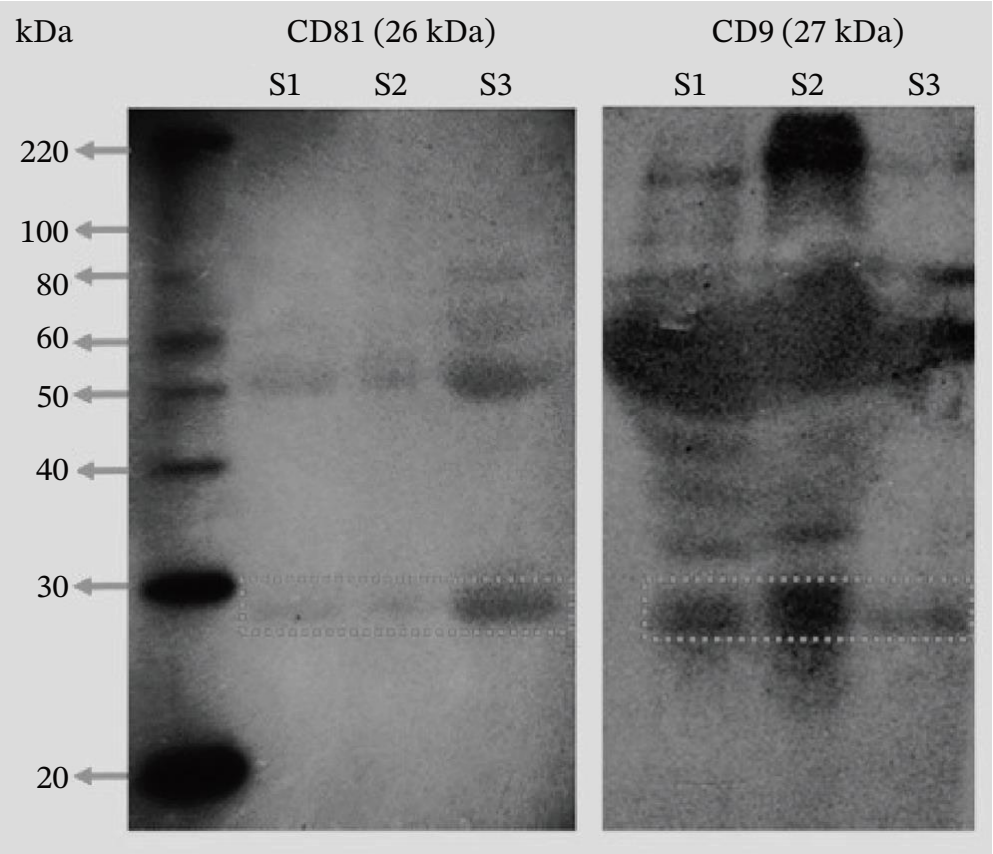
Identity of EVs by Western blot. Representative photo of tetraspanins CD81 (26 kDa) and CD9 (27 kDa). *n* = 3 different samples. S1 = Sample 1, S2 = Sample 2, and S3 = Sample 3.

Western blotting demonstrated the presence of the EV markers CD81 and CD9 (Figure 7) in the secretome samples studied.

#### 3.1.5. Identification of Cancer Markers

Using the Milliplex MAP Kit Human Circulating Cancer Biomarker Magnetic Bead, Panel 4 (Millipore, HCCB4MAG‐58K), the following results were obtained in the secretome of CSCs from both cell lines and in TNBC patients and healthy women.

**Figure 8:**
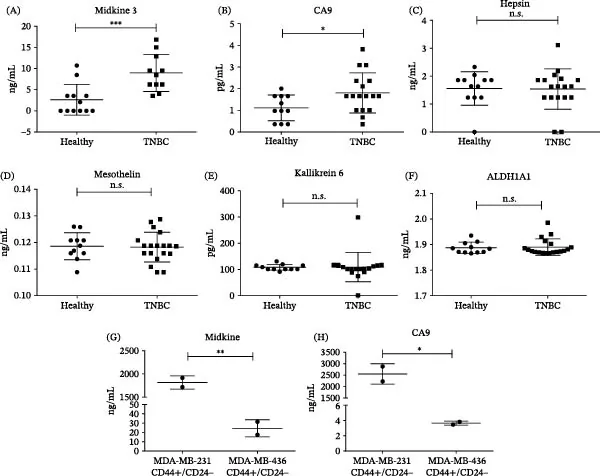
Distribution of markers in healthy women and TNBC patients (A–F). Midkine (G) and CA9 (H) values were found to be significantly higher in MDA‐MB‐231 and MDA‐MB‐436 cell lines than those found in TNBC patients. n.s = not significant;  ^∗^ *p* < 0.05;  ^∗∗^ *p* < 0.01; and  ^∗∗∗^ *p* < 0.001.

The markers that clearly distinguished between healthy and TNBC patients were midkine and CA9 (Figure 8A,B), showing significant differences. The other markers did not tend to have higher values in patients with TNBC (Figure 8C–G). However, midkine better discriminated the control group, both in vivo (Figure 8A) and in the CSCs of the two cell lines studied (Figure 8G).

## 4. Discussion

The secretome isolation method used was based on the use of Amicon centrifugal filters, which allowed a wide size distribution for the particles obtained according to the SD of the results. This provided particle recovery comparable to other isolation methods [24], allowing the quantification of the wide variability of particle sizes in the secretome. The current methods are considered to have intermediate recovery and specificity, as they recover a larger amount of nanoparticles from the secretome, along with a certain amount of protein [25], which may be limited by the use of prefiltration as performed in this study. In this context, although the secretome isolation method used in the present study is accepted for nanoparticle isolation, it is recommended to combine it with other recovery methods, and thus, efficient and standardised methods should be developed to isolate and detect EVs [26].

This work focused on the isolation of CD44+/CD24− immunophenotype CSC secretome nanoparticles from two TNBC cell lines called MDA‐MB‐231 and MDA‐MB‐436, as well as from the plasma of women with TNBC, which is heterogeneous. This is in agreement with previous studies that demonstrated a wide variability of nanoparticle size [27], which were heterogeneous and membrane‐enclosed populations, constituting EVs [28]. Furthermore, differences in the size and concentration of secretome particles between the two cell lines highlight the heterogeneity of the secretome components identified in this study, which could contribute to individual variations in disease progression and response to chemotherapy. We therefore consider it pertinent to extend these studies to TNBC.

This study showed that the frequency of particles was heterogenous, being highest in the CSC‐MDA‐MB‐231 immunophenotype, ranging from 201–500 nm (97%), followed by the MDA‐MB‐231 line with 86%, and lastly, the MDA‐MB‐436 line with 52%. These were found in medium‐sized vesicles called large EVs [25].

However, the CSC‐MDA‐MB‐436 immunophenotype had a frequency of 65% in the 0–200 nm range corresponding to small EVs or EVs, following the nomenclature for EVs proposed by Information for Studies of EVs (ISEV) [25]. It should be noted that this study used the SEC technique for EV isolation and the TRPS technique using the qNano system for characterisation, which is important to take into account when comparing these results with others that have used different isolation and characterisation techniques.

The TRPS technique used in this study with the qNano system is widely recognised, and we followed protocols described by other authors [29, 30], which allowed demonstrating the presence of particles in the secretome of the isolated cell lines, requiring small amounts of sample for analysis, as previously described by Shao et al. [31] and Kwon et al. [32], unlike other techniques used for the same purpose. Due to the high particle recovery, multiple dilutions were necessary to obtain a nonclogging nanopore, allowing for complete analysis of particle size and concentration in the secretome and a complete and detailed statistical analysis of the nanoparticles, similar to that described in previous studies [27, 28].

On the other hand, we also found differences in particle concentrations, finding a higher concentration in CSCs of the CD44+/CD24− immunophenotype, as CSC‐MDA‐MB‐231 had 1.3 × 10^10^, while the line from which they originate had 3.8 × 10^7^. CSC‐MDA‐MB‐436 had a concentration of 2.7 × 10^8^, which was higher than the L‐MDA‐MB‐436 line, which had a concentration of 9.4 × 10^7^. These values show that the CSCs‐MDA‐MB‐231 line had a higher concentration of particles than CSC‐MDA‐MB‐436. Similarly, both CSCs presented a higher concentration of particles than the cell lines from which they are derived, although they were all treated with the same cell culture protocol and production of the CM. These differences could be explained by the different cellular stress during the cryopreservation process, as demonstrated in other studies [33, 34], or by differences inherent to their own biology [35]. While the differences found in this work focused on a higher frequency and concentration of nanoparticles by CSCs with the CD44+/CD24− immunophenotype, this may be explained by the fact that these are intratumoural cells associated with chemoresistance and metastasis, qualities that require a high migratory and population growth capacity [36, 37] that could respond to a higher production and heterogeneity of particles.

It has been suggested that the quantitative and qualitative qualities of CSC secretome EVs could explain the high degree of intercellular communication that allows the transfer of large biomolecular loads between different cell types [38] and the ability to regulate the composition of the tumour microenvironment together with cytokines, growth factors, metabolites, and hormones, thus regulating the growth of surrounding cells [39–41]. There is also evidence in a highly invasive and metastatic cell line that certain subpopulations of nanoparticles in the cell secretome are more likely to trigger this type of protumourigenic activity [42], which justifies the importance of identifying the size ranges in which a greater presence of these particles is observed. All this raises the need to quantitatively and qualitatively identify nanoparticles in these cell subpopulations, such as CSCs, for better understanding of the pathogenesis of TNBC [43–45], as well as to use EVs from the secretome as carriers of antibodies directed against CSCs as therapeutic targets, as proposed by other researchers [46].

On the contrary, we consider that in this study, the expression of total proteins in the plasma secretome of patients with TNBC was higher compared to patients without a history of cancer, which suggests that neoplastic cells in patients with TNBC produce a greater amount of proteins that could be participating in the carcinogenesis of this type of breast cancer and that it is important to identify them.

Moreover, the secretome of the patients studied was analysed using a TRPS with particle sizes ranging from 150–250 nm in TNBC patients and 150–300 nm in healthy individuals. It was found that the particle size of samples from patients with TNBC and healthy individuals fell within different ranges, with a significant difference (*p*  < 0.01), suggesting that this parameter may be influenced by health status or, in the case of TNBC, by the presence of the disease. On the other hand, the use of TEM demonstrated the characteristic morphology of EV particles, while Western blotting determined the presence of the transpanins CD81 and CD9, which, according to previous work, are specific markers expressed on extracellular membrane vesicles that play an important role in intercellular communication when cancer metastasis is established [20].

To our knowledge, the data reported here have not yet been described by previous studies, and we consider these data important for future studies seeking biomarkers of CSCs in TNBC. Our results are also important for basic studies on the cell biology of CSCs in general, as differences in the size, frequencies, and concentration of particles contained in their secretomes will allow exploring new explanations for their different biological, clinical, and carcinogenic behaviours. However, it is recommended to integrate qualitative methods to identify the nature of nanoparticle subpopulations [47]. Nevertheless, in general, the quantitative values obtained in this study are comparable with the current nomenclature for the study of particles in different biological fluids such as cell culture [25].

Using the Luminex xMAP detection method with Luminex MAGPIX luminescence equipment, it was determined that the values of the markers midkine and CA9 had higher and lower values, respectively, and were significantly higher in TNBC patients than in healthy individuals. While in the secretome of the stem cells of the two lines studied the levels of midkine and CA9 were higher in TNBC patients, we postulate that both markers are circulating in TNBC patients and may be produced by CSCs that have the CD44+/CD24− phenotype. However, the levels of midkine are significantly higher. These results have not yet been described by other researchers.

Based on these results, it is important to determine the relationship between midkine and CA9 and their production by CSCs, as both molecules are related to the stem‐like characteristics of neoplastic cells. This is reinforced by the fact that the involvement of midkine—the most representative molecule of this work—in breast cancer carcinogenesis has been described by other authors, and it has been confirmed that this molecule influences breast cancer progression through the regulation of the NF‐kB pathway, favouring breast cancer cell proliferation and migration [48]. Another study has also shown that the overexpression of midkine drives epithelial‐mesenchymal transition activation and cancer metastasis [49], while a further study showed that midkine mediates cell growth, survival, metastasis, migration, and angiogenesis [43]. However, these studies do not specify the type of cells that produce midkine, and thus, the results of our study suggest that CSCs are responsible for the increased production of midkine, although another study proposed that this molecule plays a key role in maintaining the stem‐like and tumourigenic properties of glioma‐initiating cells [50]. Furthermore, analysis of the biomarkers studied using the Milliplex MAP magnetic bead kit for circulating tumour biomarkers in humans, Panel 4, established a range of 5–15 ng/mL, with no correlation observed with cancer stage; a larger sample size may be required to reach a definitive conclusion.

In this study, the molecular marker CA9 was found to have elevated levels in CSCs, while in the serum of TNBC and healthy women, the values were low. However, there was a significant difference between the two groups, indicating a relationship between CSCs and CA9 production. In this regard, we have not found any study that establishes the cellular origin of CA9, and thus, our results provide the first evidence that it is CSCs that produce this transmembrane protein. However, we should note that other studies have demonstrated the presence and involvement of CA9 in triple‐negative breast tumours [51], while others have linked it to cancer cell invasion and metastatic characteristics, as well as to the induction of stem cell–like characteristics, drug resistance, and recurrence [52], and it has even been postulated as a biomarker for breast cancer in plasma and tissues [53, 54].

In this study, we were able to differentiate the plasma secretome of women with TNBC from that of healthy women based on the presence or absence of midkine and CA9 molecules, which were shown to be produced in vitro by breast CSCs with the CD44+/CD24− phenotype. The average amount of MIDKINE found in CSC‐MDA‐MB‐231 was 1815.69 ng/mL, while 7.75 ng/mL was found in the patient’s plasma, which is 200 times more than in plasma. In the case of CA9 present in CSC‐MDA‐MB‐231, 24.87 ng/mL was found, while 1.73 ng/mL was found in plasma, which is three times more than in plasma. Therefore, we propose that they are relevant markers in this type of cancer. Since the secretome is known to originate from inside cells, which in this case were CSCs [55], the molecules detected were exclusive to women with TNBC. It can therefore be postulated that these molecules are produced by the CSCs, which, according to other researchers, should be established in order to have biomarkers that allow determining the role played by CSCs in cancer and to allow differentiation from normal stem cells, as well as identify their specific biological, molecular, and cellular characteristics as biomarkers of greater precision [17, 56]. This proposal is reinforced by a recent study by a group demonstrating that CSCs are derived from normal progenitor cells, which are influenced by midkine [57].

Our results show that midkine and CA9 are present in the secretome of TNBC stem cells with the CD44+/CD24− immunophenotype from two cell lines, as well as in the plasma of women with TNBC. They are, however, absent in healthy women, suggesting that midkine and CA9 are produced by these cells only in women with TNBC, indicating their key implication in this type of cancer and that they may play an important role in the development of metastasis and postchemotherapy recurrence in TNBC. These plasma stem cell biomarkers differ from others, such as the membrane glycoprotein CA15‐3/MUC‐1, which is not specific to this cell subpopulation [58–60].

To conclusively confirm the functional dependence of CSCs on midkine and CA9, we believe that future gene silencing studies on both markers should be conducted, particularly using the MDA‐MB‐231 cell line. Nonetheless, the limitations of this work are the small number of blood plasma samples and cell lines studied, and thus, midkine and CA9 cannot yet be conclusively established as biomarkers for CSCs in TNBC.

## 5. Conclusions

The size and concentration of the EVs in the secretome of CSCs are heterogeneous. In terms of size, EVs of 0–200 nm are more frequent in CSCs‐MDA‐MB‐436, while EVs of 201–500 nm in size and the concentration of CSCs are more frequent and higher, respectively, in CSCs‐MDA‐MB‐231.

Midkine and CA9 are present in the secretome of TNBC stem cells, as well as in the plasma of women with TNBC, but are absent in healthy women; therefore, they could be considered potential biomarkers of these cells in this type of tumor.

## Funding

This work was supported by the Sistema Nacional de Ciencia, Tecnología e Innovación Tecnológica (Grant 104‐2018‐FONDECYT‐BM‐IADT‐AV) and the Universidad Científica del Sur, Lima, Perú.

## Conflicts of Interest

The authors declare no conflicts of interest.

## Data Availability

All the data used to support the findings of this study are included in the article.
